# Interactions between the gut microbiota-derived functional factors and intestinal epithelial cells – implication in the microbiota-host mutualism

**DOI:** 10.3389/fimmu.2022.1006081

**Published:** 2022-09-08

**Authors:** Harpreet Kaur, Syed Azmal Ali, Fang Yan

**Affiliations:** ^1^ Department of Pediatrics, Vanderbilt University Medical Center, Nashville, TN, United States; ^2^ German Cancer Research Center, Division of Proteomics of Stem Cell and Cancer, Heidelberg, Germany; ^3^ Department of Cell and Developmental Biology, Vanderbilt University, Nashville, TN, United States

**Keywords:** extracellular vesicle, intestinal inflammation, intestinal epithelial cell, metabolite, probiotics, secretory product, commensal microbiota, mutualism

## Abstract

Mutual interactions between the gut microbiota and the host play essential roles in maintaining human health and providing a nutrient-rich environment for the gut microbial community. Intestinal epithelial cells (IECs) provide the frontline responses to the gut microbiota for maintaining intestinal homeostasis. Emerging evidence points to commensal bacterium-derived components as functional factors for the action of commensal bacteria, including protecting intestinal integrity and mitigating susceptibility of intestinal inflammation. Furthermore, IECs have been found to communicate with the gut commensal bacteria to shape the composition and function of the microbial community. This review will discuss the current understanding of the beneficial effects of functional factors secreted by commensal bacteria on IECs, with focus on soluble proteins, metabolites, and surface layer components, and highlight the impact of IECs on the commensal microbial profile. This knowledge provides a proof-of-concept model for understanding of mechanisms underlying the microbiota-host mutualism.

## Introduction

The human microbial community comprises more than one trillion microorganisms, including bacteria, fungus, viruses, and protozoa, which makes the number of the microbial cells almost equal to the total number of cells in human body (1, 2). The taxonomic composition of the human microbiota exhibits high interpersonal differences; however, microbial genes and metabolic modules share similar functions (3, 4). In the gastrointestinal tract of healthy adults, *Firmicutes*, *Bacteroidetes*, *Proteobacteria*, *Actinobacteria*, and *Verrucomicrobia* are the main commensal microbial phyla (5), with *Firmicutes* and *Bacteroidetes* accounting as the majority of phyla (6). In contrast to the relative abundance of commensal microbiota, the normal human gut also contains pathobiota with potential pathogenic behavior (7). Symbiotic relationships between commensal bacteria and the host are established through a variety of ways that are mutually beneficial. The commensal bacteria provide nutrients to host *via* digesting dietary components that can be used as energy sources (8), prevent the colonization of pathogenic bacteria by competitive inhibition of pathogen binding to host cells and secretion of antimicrobial compounds (9), and affect many aspects of host metabolism and physiological processes that lead to direct influence on modulating protective immune responses (10), maintaining intestinal epithelial homeostasis (11) and mediating the gut–brain axis for the function of the nervous system (12). In turn, the gut microbiota as the most diverse and populous microbial assemblage (13) is influenced by the host factors through several means. In addition to the nutritional support from the host, the composition of the intestinal microbiome is shaped and structured by the genotype of the host and factors associated with lifestyle, environmental exposure, and diseases (1). Further, increasing evidence reveals that host-derived factors participate in regulating bacterial adaptation, growth, and function (14).

The health-promoting impact of the mutualistic relationships between the gut microbiota and the host supports the therapeutic potential of the microbiota-targeting approach such as probiotics and prebiotics. Probiotics, which are beneficial commensal bacteria to host health, have shown promising outcomes in human, animal, and *in vitro* studies (15, 16). The most widely used probiotics, *Lactobacillus* and *Bifidobacterium*, have shown the high survival properties in the gastrointestinal acidic environment (17, 18). Likewise, prebiotics is non-digestible dietary ingredients that promote the survival of beneficial probiotic species (19). However, current understanding is insufficient to exploit the clinical efficacy of probiotics (20, 21). In addition to wide variations in probiotic strain selection and dosing in probiotic clinical trials, uncertain clinical outcomes result from lack of precision in host variables, including the health condition, gut microbiome profile, and diet (15), which may limit probiotic bioavailability and biopharmacology in the gastrointestinal tract. Therefore, probiotic bacteria-derived functional factors with effectiveness for promoting health are in high demand.

The first driver of the gut microbiota and host interactions occurs at the monolayer of intestinal epithelial cells (IECs). IECs contain different cell types with unique functions: enterocytes for absorption of nutrients, transport water and waste products; goblet cells for production of mucus, enteroendocrine cells for hormone secretion; Paneth cells for secretion of antimicrobial peptides (AMPs), and microfold (M) cells involved in antigen capture and presentation to immune cells (22). IECs also contribute to host immunity by secreting cytokines and chemokines (23, 24). Most importantly, IECs serve as the front line for the host to interact with the intestinal luminal factors such as the gut microbiota and their secretory products and metabolites. The mucosal barrier formed by tight junctional complexes within IECs (25) and the layer of mucus protects the host against pathogen and toxic substance invasion (26). Notably, commensal bacteria stimulate several beneficial cellular responses in IECs for intestinal development, mucosal barrier, and intestinal homeostasis (23).

Mechanisms underlying the regulatory effects of commensal bacterium-derived factors on the host and the impact of components in the gut luminal environment supported by the host on shaping the composition and function of commensal bacteria are beginning to be understood. This review will highlight updated information on the mutualistic relationships between IECs and commensal bacteria, with the focus on commensal bacterium-derived functional factors. Knowledge to elucidate the mutualism-led health-promoting outcomes can pave a new avenue for developing microbiota-targeting therapies.

## Commensal bacterium-derived factors promote intestinal epithelial homeostasis

Microbe-derived factors refer to a complex of secreted micro- and macromolecules such as products (proteins, enzymes, organic acids, and bacteriocins), metabolites (short-chain fatty acids, SCFAs), and bacterial fractions (muropeptides, teichoic acids, endo- and exopolysaccharides, and surface-layer proteins), which are naturally generated by live bacteria or made in fermentation process (27). Notably, factors produced by commensal bacteria are recognized by IECs and can induce beneficial signaling in IECs, resulting in maintaining intestinal homeostasis. IECs serve as the initial interface with the gut microbiota and a first line of defense against harmful microbes and contribute to translating commensal microbiota-elicited signals into specific cellular responses, thus can operate as the functional connection of commensal bacterial activity and hemostasis in the host (17). The beneficial effects of the interactions between commensal bacteria and epithelial cells occur not only in the gut but also in other parts of the human body. Studies have shown that commensal bacteria protect the skin against local pathogen infection (28). The importance of the interactions between commensal bacteria and IECs is reflected by the genetic evidence that the impaired recognition of commensal bacteria is associated with development of intestinal inflammatory diseases, such as many inflammatory bowel disease (IBD) susceptibility genes have been found to be involved in regulating host–microbial interactions (29, 30). Therefore, to prevent uncontrolled inflammatory responses, new strategies focused on restoring the normal balance of the intestinal ecosystem are under development (31).

### A secretory protein – p40

p40, which was originally isolated from the culture supernatant of *Lactobacillus rhamnosus* GG **(**LGG) (32), is the first recognized biologically active component of a Gram-positive commensal and probiotic bacterium, *L. rhamnosus GG* (LGG), for benefiting intestinal functional maturation and protecting IECs against inflammatory insults. Genes encoding proteins of the p40 cluster are mainly present in species related to the *L. casei, L. paracasei, L. zeae and L. rhamnosus* taxonomic group. In fact, p40 has been detected in culture supernatants of several strains of *Lactobacillus* (33–35). p40 homolog genes are also present in some species of the families Enterococcaceae and Streptococcaceae (36). C-terminal domain of p40 contains a histidine-dependent amidohydrolase/peptidase (CHAP) domain with cell wall hydrolase activity (33). Interestingly, p40 has been found to bind lipoteichoic acid (LTA) on the external surface of extracellular vesicles (EVs) released by *Lactobacillus casei BL23* (37), suggesting a manner for secretion of p40 by bacteria. It is unknown that whether there is free form of p40 section.

**Figure 1:**
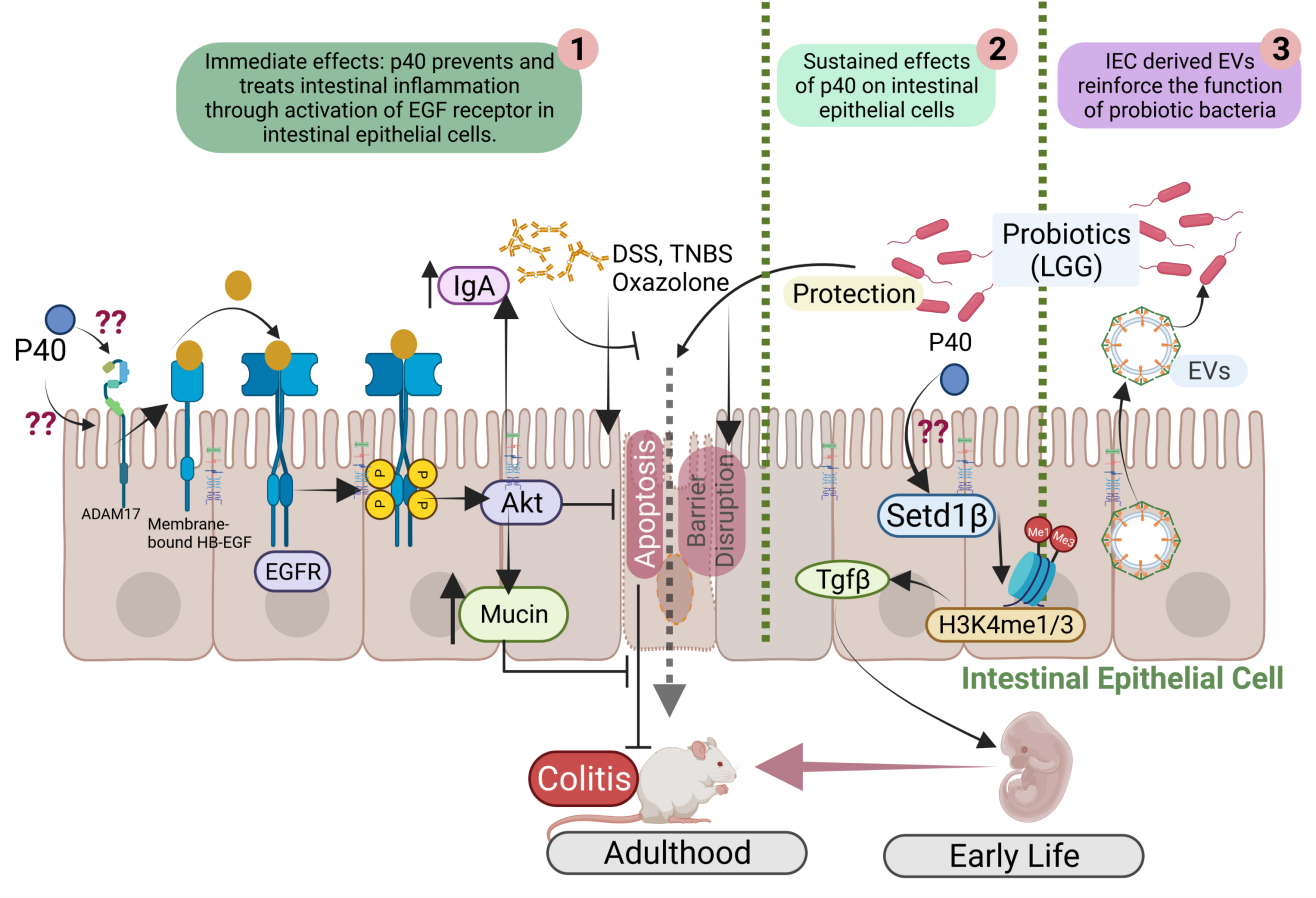
Mutual interactions between commensal bacteria and IECs. p40 is a functional factor secreted from the gut commensal bacteria. p40 has immediate effects on transactivation of EGF receptor (EGFR) and its downstream target, Akt, leading to protective responses in IECs for preventing and treating colitis (1). p40 also exerts sustained effects on IECs through upregulation of Setd1β expression and histone 3 (H3) methylation on lysine residue (H3K4me1/3) to stimulate TGFβ gene expression and protein production by IECs, thus p40 supplementation in early life prevents colitis in adulthood (2). IEC-released extracellular vehicles (EVs) communicate with commensal bacteria and promote the function of commensal bacteria (3).

Studies have revealed that p40 exerts immediate and long-lasting effects on IECs (**Figure 1**) through two distinct mechanisms and these two functions are independent. The immediately effect of p40 is through transactivation of epidermal growth factor receptor (EGFR) and its downstream target, Akt, in IECs. In addition to direct ligand binding to EGFR for its activation, EGFR can be transactivated by other pathways that stimulate A Disintegrin and Metalloproteinase (ADAM)17-triggered releasing transmembrane EGFR ligands for binding to EGFR (38). Studies found that p40 up-regulated ADAM17 catalytic activity to stimulate membrane-bound heparin binding (HB)-EGF release in human and mouse intestinal epithelial cell lines and in mice (32). The biological consequences of EGFR transactivation by p40 have been unraveled, including inhibition of proinflammatory cytokine-induced apoptosis, preserving tight junctions in IECs (39, 40), and promoting mucin production by goblet cells (41). Furthermore, transactivation of EGFR by p40 not only stimulates protective roles on IECs but also induces innate immunity: p40 upregulates gene expression and protein production of proliferation inducing ligand (APRIL) gene expression in IECs, which is a cytokine involved in B cell class switching to IgA^+^ cells, thereby increasing IgA^+^ plasma cells and IgA production (42). EGFR activation contributes to multiple protective cellular effects in colitis (43). Strong evidence indicates that p40 transactivation of EGFR in IECs contributes to ameliorating colitis in mice (44).

Furthermore, EGFR signaling is required for postnatal growth (45). p40 significantly enhanced functional maturation of the intestine, including intestinal epithelial cell proliferation, differentiation, and tight junction formation, and IgA production in early life in wild-type mice, which is mediated by transactivation of EGFR in IECs (46). These results define a mechanism of p40 regulated immediate effect on protection of intestinal epithelium through induction of ADAM17-mediated EGFR ligand release, leading to transactivation of EGFR in IECs.

Colonization of the gut microbiota in a critical window in early life enables life-long health outcomes in humans and animals (47). It has been reported that p40 supplementation in early life stimulated long-lasting effects on TGF-β production by IECs. Two outcomes of this effects have been reported: promoting induction of differentiation of regulatory T cells (Tregs) in the lamina propria of the small intestine and the colon and protecting of epithelial barrier and inhibiting proinflammatory cytokine production in IECs. One ultimate result by early p40 supplementation is to prevent colitis in adulthood (44, 46).

The long-lasting effects of p40 has been related to its epigenetic modification of IECs. Epigenetic reprogramming refers to global remodeling of epigenetic marks *via* which the host identifies the microbial signal to convert them into the long-term specific cellular signal. In the IECs and immune cells, epigenetic modification permits the gut microbiota to regulate gene expression and cellular responses (48). The COMPASS complex contains methyltransferase and adaptors to activate target gene expression by catalyzing mono- and tri-methylation of histone 3 lysine 4 residue (H3K4me1/3) at enhancer and promoter sites (49). Setd1β, a methyltransferase in COMPASS complex, has a specific function in the assembly and regulation of H3K4 mono-, di and trimethylation. p40 has been found to upregulate *Setd1β* gene expression and protein production, which mediate programming the TGFβ locus into a transcriptionally permissive chromatin state and promoting TGFβ production in IECs. Interestingly, p40 supplementation in early life, but in adulthood, could induce sustained H3K4me1/3 in IECs toward TGFβ production (44). These results establish a novel mechanism involved life-long effects on maintain homeostasis by supplementation with commensal bacterium-derived factor in early life.

### Metabolites - short-chain fatty acids and indole

The gut bacteria can metabolite a compound of phytochemicals in dietary fiber-enriched meals into SCFAs, including acetate, butyrate, and propionate. In particularly, butyrate is rapidly absorbed from the intestinal lumen and is the preferred source of energy for colonic epithelial cells (50). Regulation of epithelial barrier by SCFAs has been reported through several mechanisms. Reinforcing the epithelial barrier and enhancing wound healing through promoting the expression of the actin-binding protein synaptopodin (SYNPO) has been identified as a role of a SCFA, butyrate (51). Butyrate decreases gut permeability by enhancing tight junction protein claudin-2 upregulated expression *via* an interleukin 10 receptor subunit alpha (IL-10RA)-dependent mechanism (52). SCFA produced by the symbiotic bacteria, *Akkermansia muciniphila*, *Clostridium butyricum*, and *Faecalibacterium prausnitzii* produce SCFAs in the intestinal tract function as inhibitors of histone deacetylases (HDACs) by upregulating histone acetyltransferases activity, while possessing anti-inflammatory and epithelial barrier maintenance effects in various animal models (53). As proven, the epigenetic effect of SCFAs as inhibitors of HDACs is mostly butyrate>propionate>acetate, which results in increased levels of histone acetylation, decondensation, and relaxing of chromatin (54). *In vitro* studies have shown that SCFAs have the potential to be the intestinal protection barrier by inhibiting the enzyme Histone Deacetylase (HDAC), which has preventative effects on DNA transcription, regulates gene expression, and increases the expression of MUC2, MUC1, MUC3, and MUC4 (55). Furthermore, both butyrate and propionate upregulated MUC2 transcript expression in LS174T cells. Analysis of the MUC2 promoter indicated that an active butyrate-responsive region comprising an AP1 (c-Fos/c-Jun) cis-element is necessary for the activation of MUC2 by acetylation and methylation of histones (56). Another study has shown that SCFAs strengthen the epithelial cell tight junctions, resulting in a robust and healthy intestinal barrier. Butyrate maintains and enhances the transepithelial electrical resistance (TEER) in Caco-2 cells that is mediated by AMP activated protein kinase (AMPK) (57).

SCFAs such as butyrate and propionate play significant roles in immunity through regulation of IECs and immune cells, such as T cells, macrophages, and dendritic cells. A report showed that SCFAs and a high-fiber diet were able to induce vitamin A metabolism in epithelial cells and CD103^+^ DCs and this was associated with enhanced Foxp3 expression in T cells (58). DCs have been found to play a significant role in the initiation of IgA production in the gut in response to SCFAs. Metabolite-sensing mammalian G protein-coupled receptor (GPR43) in DCs mediated the acetate induction of intestinal IgA response to microbiota, in that GPR43 knock out mice showed lower IgA production and decreased numbers of IgA+ B cells in the intestines (59).

In addition to normal IECs, SCFA plays roles in inhibition of colorectal cancer cell growth. Major anti-proliferative activity of SCFAs is associated with butyrate, acetate, and propionate (with acetate and propionate requiring higher concentrations to be as effective as butyrate). These SCFA compounds can promote apoptosis responses in colon cancer cells by influencing mitochondrial permeability potential, producing reactive oxygen species (ROS), and activating caspase-3 (27).

Indole and its derivatives are derived from the metabolism of tryptophan by the gut bacteria containing tryptophanase such as *Lactobacillus reuteri* and Clostridium sporogenes. The absorption of indole and its derivatives (Indole 3-propionic acid (IPA), indole-3-ethanol (IEA), and indole-3-acetaldehyde (IAAld) through the intestinal epithelium is due to the ability to freely diffuse through lipid membranes (60). Indole and its derivatives support intestinal immune homeostasis through activating aryl hydrocarbon receptor (AhR) to protect the intestinal tight junctional barrier. The activation of the AhR pathway in IECs is vital for protecting the stem cell niched and maintaining intestinal barrier integrity (61). The pregnane X receptor (PXR) is a physiologic regulator associated with gut permeability (62). IPA has been found as a ligand for epithelial PXR, and the administration of IPA can up-regulate tight junction protein-coding mRNAs and enhance the expression of claudins and occludins (63). Recent studies have showed that IEA, IPA, and IAAld contribute to maintaining the integrity of the intestinal tight junctional barrier in an AhR-dependent manner and alleviating dextran sodium sulfate (DSS)-induced colitis in mice (64). Indole and its derivatives enhance IL-10 expression through aryl hydrogen receptors (AhR) activation and promote IL-10 signaling which is linked with barrier function. Indole-3-aldehyde (IAld) increases epithelial cells proliferation and upregulates the differentiation of goblet cells, intestinal barrier integrity and downregulates the systemic inflammation caused by aging in geriatric mice. This effect increases the expression of the cytokine IL-10 *via* AhR but does not depend on the type I interferon or IL-22 signaling (65). The activation of AhRs leads to lL-22 transcription, which can further increase the expression of antimicrobial peptides and improve colonization resistance against *Candida albicans* in the gastrointestinal tract (66).

Overall, these studies support the feasibility of commensal bacterial metabolites as a strategy to promote mucosal barrier and intestinal homeostasis. Empirical modulation of the microbiota using probiotics can increase SCFAs and indole-producing bacteria for the maintenance of epithelial barrier integrity in inflammatory models (67). Thus, supplementation with specific probiotics for beneficial metabolites formation could provide new avenues to manage disease activity.

### Surface layer components - surface layer protein, exopolysaccharide, peptidoglycan, and lipoteichoic acid

Bacterial surface layers contain ubiquitous proteins structures that are abundant in Gram negative and Gram-positive bacteria. In Gram-positive bacteria, the S-layer lattice is generally composed of a single protein and is attached to peptidoglycan-bound secondary cell-wall polymer by non-covalent interactions (68). As the outermost structure of the cell, the surface layer lattice is generally considered to be the first bacterial components that have a direct interaction with host cells. The effects of cell surface molecules are diverse and have been shown to play roles in many bacterial functionalities, such as adhesion to the host cells, strengthening of the gut barrier integrity, pathogen exclusion, stimulation of the host mucosal system to improve mucus production, and secretion of defense molecules such as β defensins (69).

Surface layer protein A (SPA-A) derived from *Lactobacillus acidophilus* NCK2187 binds to the C-type lectin SIGNR3 and initiates regulatory signals, leading to maintenance of healthy gastrointestinal microbiota, protecting gut mucosal barrier function, and prevention of colitis (70). Recent study revealed that SLP of *Lactobacillus acidophilus* NCFM strain led to a reduction in myeloperoxidase activity and TNF-α expression whereas significantly increased the IL-10 levels. The administration of these surface proteins significantly reversed the histopathological damages induced by the colitogens and improved the overall histological score in TNBS colitis mice model (71). Results from *in vitro* studies indicated that purified SLPs from *L. plantarum* exert a protective effect on Caco-2 cells infected with EPEC by increasing their transepithelial resistance (TEER) and down-regulating their permeability (72). Further, *L. acidophilus* contains three different SLPs, SLP-A, SLP-B, and SLP-X, which interact with pattern recognition receptors (PRRs) in IECs and modulate the immune response. *L. acidophilus* SLPs decrease interleukin (IL-8) secretion in Caco-2 cells stimulated by *S. typhimurium* (73).

Exopolysaccharide (EPS) are metabolic by-product of microorganisms (74). EPS consists of homopolysaccharides (HoPS) or heteropolysaccharides (HePS), depending on the main chain composition and mechanisms of synthesis. The most HePS-producing bacteria are *Lactobacillus, Lactococcus*, *Streptococcus* and *Enterococcus* strains frequently isolated from fermented dairy products and the human gastrointestinal tract (GIT), whereas most HoPS are produced by *Lactobacillus, Leuconostoc, Pediococcus, Streptococcus*, and Weissella strains present in animal GIT, vegetables and fermented beverages (75). EPS-1 contributes to maintaining the intestinal barrier integrity against the disruption by lipopolysaccharide (LPS) in Caco-2 monolayer mediated by enhancing. the expression of tight junction. On the transcriptional level, LPS-decreased expression of several tight junction genes was inhibited by *S. thermophilus*-derived HePS *in vivo* (76). Further, the role of EPS in epithelial adhesion of commensal bacteria makes EPS of particular interest for preventing adhesion of pathogenic bacteria. It has been demonstrated that EPS produced by *Lactobacillus paracasei subsp. Paracasei* BGSJ2 plays an essential role in the prevention of adhesion of *E. coli* to Caco-2 cells (77). Further, the immunoregulatory effects of EPS on IECs are supported by the fact that EPS activates C-type lectin receptors on IECs to elicit an immunological response. Upon stimulation by EPS, IECs secrete several cytokines and chemokines, including interleukins, TNF, growth factors, and beta-defensins (78). Therefore, IECs play an essential role in the recruitment of dendritic cells, which are responsible for controlling both innate and acquired immunological responses (79). In addition, EPS has shown to mitigate experimental colitis, improving mucosal barrier function, and modulating gut microbiota composition (80–82). Moreover, recent studies have suggested that applying EPS from lactic acid bacteria to the skin enhances skin health and proven to be aid in gastrointestinal wound healing in different *in-vitro* and *in-vivo* studies (83, 84). It would be advantageous to consider EPS as a feasible prebiotic choice for therapeutic purposes due to its two feathers: EPS is indigestibility to the host cells, thus, arriving in the colon intact and consumed by specific gut microbiota in the colon (85).

Peptidoglycan (PGN) is a large polymeric molecule present in the Gram positive and Gram-negative bacterial cell wall. The basic overall structure of PGN is conserved between different organisms, but there are backbone and crosslinking modifications that increase the variability among the bacterial species. PGN in probiotic bacteria undergo variety of modifications in the sugar structure including deacetylation, O-acetylation, and N-glycosylation, which create the differences in sugar structure leading to the alteration in the properties of the cell wall (86). There are different receptors to detect the peptidoglycan or its fragments, for example, the innate immune system can detect PGN through peptidoglycan recognition proteins (PGLYRPs) (87), which are mostly expressed in eosinophils and neutrophils and could potentially act on inflammation (88). In the intestinal lumen, PGN-contained cell wall fragments can be released from commensal bacteria after digestion by Paneth cell-derived lysozyme. It has been suggested that PGN can be absorbed by crypt-based immature intestinal epithelial cells and in transported over the intestinal epithelium (89). Functional analysis has shown that PGN secreted by *Lactobacillus* and *Bifidobacterium* enhanced the expression of tight junction proteins, including claudins, occludin, and ZO-1 and improved the integrity of the gut barrier *via* Toll-like receptors 2 signaling (90, 91).

Lipoteichoic acid (LTA) is one of the major cell wall components of Gram-positive bacteria that can be considered the pivotal components for immunomodulating effects (92). In addition to its immunoregulatory effects, such as LTA from *L. casei* YIT9029 and *L. fermentum* YIT0159 cause TNF-α production in macrophages through TLR2 receptors (93), LTA from *Lactobacillus plantarum* confers anti-inflammatory responses in porcine intestinal epithelial cell line, IPEC-J2 (94).

This evidence supports the note that commensal bacterial surface components act through evolutionary-optimized mechanism that targets IECs to benefit intestinal homeostasis.

## IECs modulate the composition and function of the gut microbiota

The microbiota-host interactions result in a mutually advantageous setting that provides a nutrient-rich environment favorable to microbiota development and survival. It is well-known that microbiota composition is mostly influenced by host genetics and environmental factors, such as diet, nutrient availability, immunological responses, and disease states (95, 96). A recent study has demonstrated the contribution of IECs to shape the composition of the gut microbiota. The lack of MHC class II in IECs resulted in the decrease in microbial-bound IgA, regulatory T cells, and immune repertoire selection, which is associated with the increase in interindividual microbiota variation and altered proportions of two taxa in the ileum. This evidence suggests that MHC class II in IECs regulates the microbiota composition (97). Paneth cells in the intestinal epithelium are the primary source of lysozyme that directly encounters commensal bacteria. It has been reported that luminal lysozyme abundance determines the composition of mucolytic microbiota in the gut and regulates mucosal inflammatory responses (98). These findings reveal the specific molecules in IECs that are involved in shaping the gut microbial community.

Regarding the impact of IECs on the function of the gut microbiota, a study has revealed that a cellular structure, extracellular vesicles (EVs) released by IECs mediate trans-kingdom interactions and regulation of the function of the gut microbial community. EVs are the membrane-bound vesicles secreted through multivesicular bodies and are comprised of complex cargos including lipids, proteins, and nucleic acids. EVs are important messengers for the intercellular communication among mammalian cells (99). The first action of IEC-released EVs on the gut microbes was discovered to inhibit growth of pathogens (100, 101). Recent studies have unraveled novel effects of EVs released by IECs on promoting the function of commensals (**Figure 1**). As a commensal bacterial model, protein cargos in IEC-released EVs were found to be transferred to LGG, suggesting that EVs can serve as a communication approach between LGG and IECs. Further, IEC-released EVs stimulate production of functional factor by LGG (102). Remarkably, HSP90 in EVs has been shown to contribute the increase the function of LGG (102). HSP90 is highly conserved from bacteria to mammals and displays functional overlaps in protein folding, enabling the stability and transportation of client proteins (103, 104). Client proteins of HSPs in *Lactobacilli* regulate growth, metabolism, transport functions, and protein synthesis under normal and stress conditions (105). Therefore, this finding provides knowledge for mechanistic understanding of the impact of the host on the functional aspects of the gut microbiota.

## Discussion

There are several challenges in the research area of the gut microbiota-derived functional factors. Elucidating the potential of commensal bacterium-derived functional factors in treatment and prevention of diseases, such inflammatory bowel disease (IBD), including ulcerative colitis (UC) and Crohn’s disease (CD) has high clinical relevance. IBD is caused by inappropriate immune responses to the intestinal microbiota in genetically susceptible individuals, leading to autoimmune damage of the intestinal barrier and chronic inflammation (106). IBD is associated with an increased risk of development of colorectal cancer (CRC). Dysbiosis serves as a risk factor or/and a consequence of inflammation and inflammation-associated carcinogenesis. Studies showed that qualitative and quantitative alteration in gut microbiota is highly associated with the abnormal immune responses and thus influence the course and development of IBD (107, 108). Moreover, the concentration of butyrate in IBD is considerably lower than in healthy controls, indicating that the dysbiosis has likely produced metabolic changes (109). Although there is evidence to show that specific intestinal microbes are associated with CRC development and progression, the mechanisms through which the abnormal microbial community mediates CRC development remains unclear. *Enterotoxigenic Bacteroides fragilis* (ETBF) can raise levels of chemokine L20 and prostaglandin E2 in intestinal epidermal cells; prostaglandin E2 plays a vital role in proliferation and enhances the secretion of IL-17 and related factors secreted by Th17 cells, leading to the development of inflammation-related CRC (110). Further, microbial products are sensed by Toll-like receptors, which trigger MyD88-mediated production of IL-23 proinflammatory cytokine which activates IL-17a, IL-6, and IL-22 release and thus promotes CRC development (111, 112). Dysbiosis related to CRC aids its progression *via* different pathways, such as driving inflammatory response, inducing DNA damage, stimulating cell and causes microbial homeostasis in specific microbiota. The imbalance of the gut microbial profile promotes functions associated with cancer such as uncontrolled cell proliferation and the loss of apoptosis. Moreover, differences in the species of gut microflora during tumorigenesis can be used as a biomarker and diagnostic tool for CRC (113).

Recent advances defining the protective effects of commensal bacterium-derived functional factors raise the theoretical possibility for alleviating the epithelial damage by commensal bacterium-derived functional as adjunct therapies for current IBD treatments that are focused on functional failure inhibition of proinflammatory responses.

The efficacy of probiotics in clinical applications is poorly understood (20, 21). Probiotics exert beneficial effects through multiple mechanisms and modalities (114–117). Approaches to enhance the action of probiotics, including increasing production of functional factors, will broaden the therapeutic applications of probiotics. Ongoing work towards comprehensively understanding of the host impact on the microbiota will likely open new avenues towards developing approaches for increasing the efficacy of probiotics for clinical application and generating next-generation probiotics.

## Author contributions

HK, SA, and FY wrote and revised the manuscript. All authors contributed to the article and approved the submitted version.

## Funding

This work was supported by National Institutes of Health (NIH) grant R01DK081134-12 (FY) and the Crohn’s & Colitis Foundation Senior Research Award (FY).

## Conflict of interest

The authors declare that the research was conducted in the absence of any commercial or financial relationships that could be construed as a potential conflict of interest.

## Publisher’s note

All claims expressed in this article are solely those of the authors and do not necessarily represent those of their affiliated organizations, or those of the publisher, the editors and the reviewers. Any product that may be evaluated in this article, or claim that may be made by its manufacturer, is not guaranteed or endorsed by the publisher.
